# Supplementary material for: Assessment of the Current Status of Potyviruses in Watermelon and Pumpkin Crops in Spain: Epidemiological Impact of Cultivated Plants and Mixed Infections
Source: Plants (Basel). 2021 Jan 12;10(1):138. doi: 10.3390/plants10010138 (PMC7827711; doi:10.3390/plants10010138)
Supplement: Supplementary file 1 [file plants-10-00138-s001.pdf]

Supplementary information

Table S1. Amino acid changes through each ORF from the complete genomes of MWMV-ZuM10 and WMV-MeWM7 isolates. Each aa change is based on their non-synonymous mutations, which were analysed by the sequence comparison with the corresponding Sq10 1.1 (MWMV type) and Vera (WMV) isolates.

Virus/ORF	MWMV-ZuM10	WMV-MeWM7
**P1**	K197E; T262I; G348S; S363L	K34E; M153V; K187N; T274A
**HC-Pro**	-	F8I; S10A; K91E; G96R; P110S; L314F; L348F; L394F
**P3**	P857S; Y872C	I2V; N30D; N206S; Y215H; I286F; G307E; K335M
**6K1**	-	S43G
**CI**	-	S249N; N291T
**6K2**	-	-
**Nia-VPg**	N2366I	E109G; S153F
**VPg-Pro**	-	M54I
**Nib**	V2423A	I42L; V434I; R482K; N505S
**CP**	-	P40Q; A41I; D21N; A52N; V58E; I243M
